# Running on the Edge: Rupture of the Tibialis Anterior Muscle During an Ultramarathon

**DOI:** 10.7759/cureus.31930

**Published:** 2022-11-27

**Authors:** João Santos-Faria, João N Malta, Alexandra P Coelho, João P Branco

**Affiliations:** 1 Physical Medicine and Rehabilitation, Centro Hospitalar e Universitário de Coimbra, Coimbra, PRT

**Keywords:** ankle dorsiflexion, ultramarathon, rehabilitation, muscle rupture, tibialis anterior muscle

## Abstract

A 47-year-old male ultramarathon runner presented with persistent discomfort in the anterior region of the left leg. The patient reported a snapping sensation in his left leg four weeks earlier while running an ultramarathon, followed by ecchymosis and functional impairment the next day. Physical examination revealed a palpable area of fibrosis in his anterior left leg. Ultrasound of the left leg identified a partially organized rupture of the distal third of the tibialis anterior muscle. The rupture had an extension of 36 x 10 x 27mm with associated muscle edema. The patient was treated non-surgically with a customized rehabilitation program and later returned to ultramarathon running. This case illustrates the importance of proper differential diagnosis and individualized rehabilitation programs to achieve optimum clinical and functional results.

## Introduction

Tibialis anterior muscle ruptures are rarely seen in clinical practice [1]. This muscle is in the anterior compartment of the leg, originating from the upper two-thirds of the tibia and the interosseus membrane. It becomes a tendon in the distal third of the leg and inserts into the medial cuneiform and the base of first metatarsal bone of the foot. It is innervated by the deep peroneal nerve and contributes to 80% of ankle dorsiflexion. Extensor hallucis longus, extensor digitorum communis and peroneus tertius muscles contribute to the rest [1,2].

An acute muscle rupture during running activities is rare but can lead to severe impairment [1]. We report a case of tibialis anterior muscle rupture during an ultramarathon, which was completed with success despite this fact.

## Case presentation

**Figure 1:**
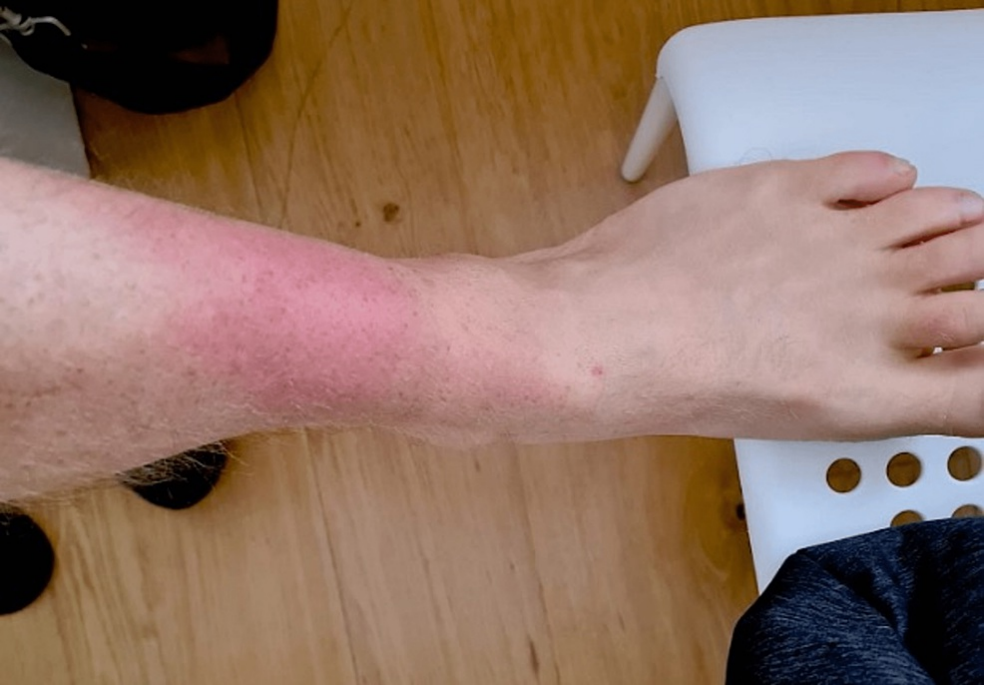
Distal area of ecchymosis of the anterior region of the left leg one day after the muscle rupture

A healthy 47-year-old ultramarathon runner with no prior medical problems other than occasional pain in the anterior left leg, was referred to a Physical Medicine and Rehabilitation consultation due to persistent discomfort in this region. He explained that four weeks prior, during an ultramarathon, he had a sudden snapping sensation in his anterior left leg. This happened in the 50^th^ kilometer of the course while he was running uphill, but he completed the total distance (140 kilometers) with no complaints other than pain, with only minor changes in his running strategy, namely stopping more frequently. The next day, he consulted a physiotherapist due to ecchymosis in the anterior left leg (Figure 1) and adjusted his physical activity accordingly during that week. There were no other interventions, and he resumed running. Before the injury, the patient had a Tegner Activity Scale (TAS) score of seven, which decreased to a level of six after the muscle rupture.

At the time of injury, the patient reported a six out of 10 on the Visual Analogic Scale (VAS) for pain. Meanwhile, at the time of observation in Physical Medicine and Rehabilitation consultation, this had reduced to three out of 10. On examination, he had a palpable region of fibrosis in his anterior left leg, with no tenderness along the course of the tibialis anterior muscle. Power in dorsiflexion and inversion was comparable bilaterally. There were no noticeable changes to report in the foot examination bilaterally and in the gait pattern.

**Figure 2:**
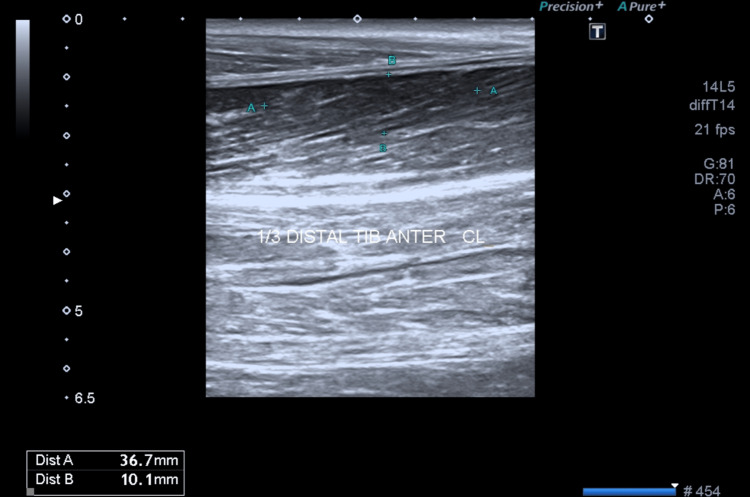
Ultrasound of the left leg showed a rupture of the distal third of the tibialis anterior muscle with an extension of 36 x 10 x 27mm

A left leg radiograph, performed one month after the ultramarathon muscle rupture, did not show any signs of periosteal changes suggestive of a stress fracture. An ultrasound was performed on the left leg (Figure 2), which showed a partially organized rupture of the distal third of the tibialis anterior muscle with an extension of 36 x 10 x 27mm associated with muscle edema. No other changes were noticeable. Further investigation, including magnetic resonance imaging, was not performed since the diagnosis had been already confirmed by ultrasound.

The patient started rehabilitation in our hospital and completed 15 sessions of a customized rehabilitation program, which is explained in Table 1.

**Table 1:** Description of the customized rehabilitation program

Rehabilitation Program (2 to 3 session/week)
Massage of the fibrotic tissue;
Muscle strengthening started with concentric exercises of the lower leg in closed kinetic chain, at 60% of 1-repetion-maximum, 10 repetitions of each exercise;
Progression to eccentric exercises in open kinetic chain;
Therapeutic ultrasound (Pulsed, frequency of 3MHz, intensity of 1W/cm^2^, for 10 minutes);
Technical gesture reeducation;
Proprioceptive training;
Cryotherapy with ice bag for 10 minutes applied to the site of injury

The patient continued running despite the rupture. He completed the rehabilitation program with success and was pain-free (0 out of 10, according to VAS) at the last medical appointment. No deficits were noticed when compared to the contralateral side three-months after the rupture. The region of palpable fibrosis was noticeably better. No other changes were found. Four months after the injury, his performance returned to pre-injury level (TAS score of seven), and he completed an ultramarathon of 180 km. 

## Discussion

The tibialis anterior muscle is the strongest dorsiflexor of the foot. It is a muscle in the anterior compartment of the leg which is primarily responsible for dorsiflexion at the ankle joint [1,2]. Closed rupture of the tibialis anterior is an uncommon injury. A 2015 systematic review found only 87 cases of tibialis anterior tendon rupture, with the largest series accounting only for 19 cases [3]. Ruptures typically occur in men between the ages of 50 and 70 years. In most cases, the muscle ruptures during supination of the ankle joint and sudden plantarflexion against resistance [4]. Muscles rarely rupture under normal circumstances, which means there is usually some sort of degenerative process involved. More commonly, ruptures occur after minor trauma suggesting pre-existing changes and are classified as acute-on-chronic ruptures [5]. Several other conditions predispose to rupture, including gout, rheumatoid arthritis, systemic pathologies like diabetes, and the use of steroids [1]. Reports of rupture have also been documented in sports like skiing and fencing [6].

According to Brunnel and McMaster, there are four different locations for a musculotendinous rupture of the anterior tibial muscle. In order of frequency, these are the insertion (e.g., avulsion), the musculotendinous junction, the muscle belly, and the origin [7]. Following a rupture, patients usually complain of gait problems, especially slap foot or drop foot, due to insufficient dorsiflexion and supination [8]. However, it is sometimes the case that the correct diagnosis is delayed because of mild symptoms or because of misdiagnosis [1]. Ouzouninan et al. presented a series of 12 patients with anterior tibial tendon rupture, with 42% reporting pain that resolved quickly. In some cases, a tendon gap was palpable. Most patients were able to fully dorsiflex the foot using the remaining functioning muscles [9]. In this case, this seems to be the reason why the athlete was able to complete the ultramarathon. On physical examination, a complete rupture reveals a defect, which is often visible. Fresh injuries are marked by ecchymoses and tenderness at the site [10].

Although the diagnosis can be established in most cases with physical examination, ultrasound and magnetic resonance imaging may be helpful where there is uncertainty about the diagnosis. They can also help characterize the rupture [9,10]. Differential diagnosis must include peroneal nerve injury, neoplasia, compartment syndrome, local peritendinitis, stress fracture, and a herniated L4/L5 disk [1].

The treatment approach to anterior tibial muscle rupture depends on the clinical presentation and patient characteristics [9]. Usually, young patients with acute ruptures are referred to surgical treatment with favorable outcomes. On the other hand, patients with chronic ruptures, who usually seek medical attention later and are typically older, are referred to non-operative treatment. In cases of drop-foot, an orthosis may be prescribed. When there is pain, pharmacological and nonpharmacological options are available [1,3,5,9]. Even though we could not find any guidelines or studies that reported the benefits of physical therapy in this subset of patients, we applied commonly accepted rehabilitation principles to manage this case. 

Complications such as calcification, areas of fibrosis, and the risk of re-injury are major concerns in the follow-up of these patients [11,12]. It's important to emphasize the importance of an individualized muscle-strengthening program to address the impact on daily activities and participation in sports [12]. 

Tendinopathy, which was probably the cause of the patient's anterior leg pain, is an established risk factor for muscle rupture, and these ruptures are classified as acute-on-chronic [5]. In this case, where the patient did not seek medical attention until weeks after the rupture, the remaining ankle dorsiflexors compensated for the partial rupture of the tibialis anterior muscle, allowing the patient to remain physically active with some minor limitations. To the best of our knowledge, this is the first case report of a tibialis anterior muscle rupture while running.

## Conclusions

The tibialis anterior muscle is the strongest dorsiflexor of the foot, and its function is essential in daily life and sports activities. A snapping anterior sensation accompanied by weakness in dorsiflexion is suggestive of tibialis anterior muscle rupture. Acute loss of function of the tibialis anterior muscle may be well tolerated in a trained athlete due to compensation by the remaining foot dorsiflexors. Physical examination is often enough to diagnose this condition. However, ultrasound and magnetic resonance imaging can be important to help confirm the diagnosis and characterize the rupture. Treatment of tibialis anterior muscle rupture can be surgical or nonsurgical, depending on presentation and patient characteristics.

## References

[1] Anagnostakos K, Bachelier F, Fürst OA, Kelm J (2006). Rupture of the anterior tibial tendon: three clinical cases, anatomical study, and literature review. Foot Ankle Int.

[2] Scheller AD, Kasser JR, Quigley TB (1980). Tendon injuries about the ankle. Orthop Clin North Am.

[3] Christman-Skieller C, Merz MK, Tansey JP (2015). A systematic review of tibialis anterior tendon rupture treatments and outcomes. Am J Orthop (Belle Mead NJ).

[4] Otte S, Klinger HM, Lorenz F, Haerer T (2002). Operative treatment in case of a closed rupture of the anterior tibial tendon. Arch Orthop Trauma Surg.

[5] Markarian GG, Kelikian AS, Brage M, Trainor T, Dias L (1998). Anterior tibialis tendon ruptures: an outcome analysis of operative versus nonoperative treatment. Foot Ankle Int.

[6] Gwynne-Jones D, Garneti N, Wyatt M (2009). Closed tibialis anterior tendon rupture: a case series. Foot Ankle Int.

[7] McMaster PE (1933). Tendon and muscle ruptures: clinical and experimental studies on the causes and location of subcutaneous ruptures. JBJS.

[8] Beyerlein J, Imhoff AB (2004). Tibialis-anterior-Ruptur. Fußchirurgie.

[9] Ouzounian TJ, Anderson R (1995). Anterior tibial tendon rupture. Foot Ankle Int.

[10] Jerome JT, Varghese M, Sankaran B, Thomas S, Thirumagal SK (2008). Tibialis anterior tendon rupture in gout--case report and literature review. Foot Ankle Surg.

[11] Alessandrino F, Balconi G (2013). Complications of muscle injuries. J Ultrasound.

[12] Kauwe M (2017). Acute Achilles tendon rupture: clinical evaluation, conservative management, and early active rehabilitation. Clin Podiatr Med Surg.

